# Prevalence of Depression and Associated Factors among Diabetic Patients in an Outpatient Diabetes Clinic

**DOI:** 10.1155/2019/2083196

**Published:** 2019-01-15

**Authors:** Zahra D. Khan, Janet Lutale, Sibtain M. Moledina

**Affiliations:** Department of Internal Medicine, Muhimbili University of Health and Allied Sciences, P.O. Box 65001, Dar es Salaam, Tanzania

## Abstract

Despite adequate treatment for diabetes, it is estimated that 15%- 20% of people with diabetes are struggling with a moderate to severe form of depression daily. Little is known about depression in diabetes in East Africa, particularly in Tanzania. The study is aimed at determining the prevalence of depression and associated factors among patients with diabetes. A descriptive cross-sectional study was carried out at the diabetes clinic of Muhimbili National Hospital. The 9-item Patient Health Questionnaire (PHQ 9) scale was used to assess presence of depressive symptoms among diabetes patients at the clinic. In addition, patient's sociodemographic and clinical characteristics were obtained and analysed for their association with depression. A total of 353 participants were recruited, of whom 229 (64.9%) patients were female and 156 (44.2%) were aged between 41 and 60 years. The overall prevalence of depression among diabetes patients at the diabetes clinic was 87%. Most (56.7%) had minimal depression, 22.1% had mild depression, and 8.2% had moderate depression. None had severe depression. Factors independently associated with a diagnosis of mild to moderate depression were being on insulin therapy and being a current smoker. There was a high prevalence of depression in this diabetic population. Majority of patients had minimal depression but about 30% had either mild or moderate depression. A holistic approach that focuses on the identification and management of depression among patients with diabetes is recommended.

## 1. Introduction

Depression is a state of low mood and aversion to activity that can affect a person's thoughts, behaviour, feelings, and sense of wellbeing. Depressed people can feel sad, anxious, empty, hopeless, helpless, worthless, guilty, irritable, or restless [1].

Globally, depression is the second-leading cause of disability, and diabetic patients have been reported to be more likely to develop depression than nondiabetes people. It is estimated that 15%-20% of people with diabetes are struggling with depression, more likely moderate to severe form of depression [2].

The etiology of depression in diabetes is not known but is probably complex; and genetic, biological, and psychological factors remain to be potential contributors [3]. Several neurotransmitter and neuron-endocrine defects have been identified to be common for both depression and diabetes, adding to etiological speculations [4].

Diabetes and depression are separate entities and are by themselves major health problems in the world. The coexistence of depression in people with diabetes might be associated with poor adherence to treatment, poor metabolic control, higher complication rates, decreased quality of life, increased healthcare use and cost, increased disability and loss of productivity, and increased risk of death.

Majority of studies that have investigated the burden of depression in diabetes mellitus (DM) have been carried out in high income countries but little is done in sub-Saharan Africa especially in East Africa [4–6]. A study survey, carried out in 60 countries across the world, using ICD-10, a one-year prevalence of depressive episode in people with diabetes was 9.3% as compared to 3.2% in people without diabetes [7].

The International Diabetes Foundation (IDF) recommends periodic assessment and monitoring of depression among patients with diabetes due to the high risk of depression in this patient group.

In Tanzania, there is no published data on the prevalence of depression among patients with diabetes. It is our hope that this study will help to expand our understanding of the extent and the relationship of depression to diabetes. Hence, this study is aimed at determining prevalence of depression and associated factors among patients at a diabetes clinic in Dar es Salaam, Tanzania.

## 2. Materials and Methods

The study was carried out at the diabetic clinic at Muhimbili National Hospital (MNH). MNH is the main referral and a teaching hospital in Tanzania. Patients were recruited from the MNH diabetes clinic on the allotted clinic days (Mondays, Wednesdays, and Thursdays) every week. The Monday clinic is for young patients mainly type 1 diabetes patients, while the Wednesday clinic caters for private and insured patients (both type 1 and type 2), and the Thursday clinic is for adults, mainly type 2 diabetes patients. On average, about 20 to 25 patients are seen on each clinic day. Patients were recruited from the clinics on Wednesdays and Thursdays. Approximately 30 patients were recruited per week for a total of three months of data collection. Inclusion criteria in this study were all patients diagnosed with diabetes at least one year before the start of the study, who were able to understand and respond to the questionnaire items and willing to participate in the study. The study excluded those patients who were currently being treated for depression. Sampling was done using systematic sampling technique from the registry in the computer, where every second patient was dropped (i.e., 2nd, 4th, and 6th).

A structured questionnaire was used to get social demographic information such as age, sex, marital status, level of education, occupation, and history of current smoking and/or alcohol use. Information was also obtained on type and duration of diabetes, current pharmacological medications, and whether on treatment for depression. Participants were asked to report all the medications that they used on chronic basis; the data reported by the participants regarding their medications was validated through their hospital medical records. Interviews were carried out by medical doctors on duty in the diabetes clinic. The questionnaire was explained to all interviewers and other details were discussed to ensure uniformity of data collection by all interviewers. In this study depression was defined using the validated PHQ-9 tool. The study categorized and graded participants with depression as follows: those with a score of 0 had no depression, 1-4 minimal, 5-9 mild, 10-19 moderate, and 20-27 severe depression.

The data obtained was entered onto Statistical Package for Social Science (SPSS) version 20 for analysis. Descriptive statistics were carried out for all variables and expressed as mean ± SD. Chi-square test was used to determine associations between variables and a p value of <0.05 was considered statistically significant. Variables that were found to be significantly associated with mild to moderate depression (p <0.05) were entered into the multivariate regression model.

Ethical clearance to carry out the study was obtained from the ethical review boards of Muhimbili National Hospital and Muhimbili University of Health and Allied Sciences. All patients were recruited into the study after an informed consent. Data obtained during the study was kept anonymous. All patients received the same standard of treatment regardless of their eligibility or choice to participate in this study or not.

## 3. Results

There were 353 participants recruited into this study. Nearly half (44.2%) were between 41 to 60 years of age with a mean age of 48.6 ± 18.0 years. The majority were females (64.9%) and had type 2 diabetes (79.6%) (Table 1).

**Figure 1:**
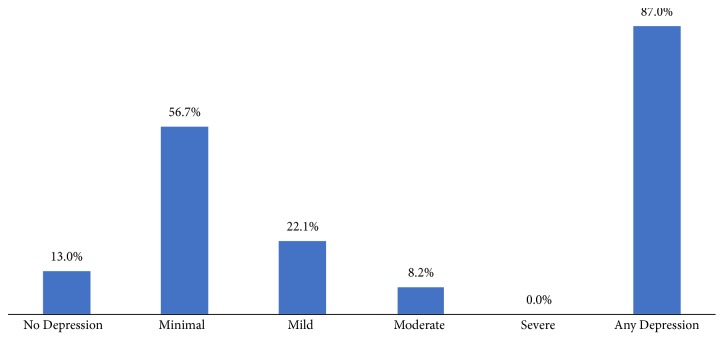
Prevalence of depression among diabetes patients.

The overall prevalence of any level of depression in this study was 87%. Of the 307 depressed patients, 56.7% had minimal depression, 22.1% had mild depression, 8.2% had moderate depression, and 13% had no depression (Figure 1).

**Figure 2:**
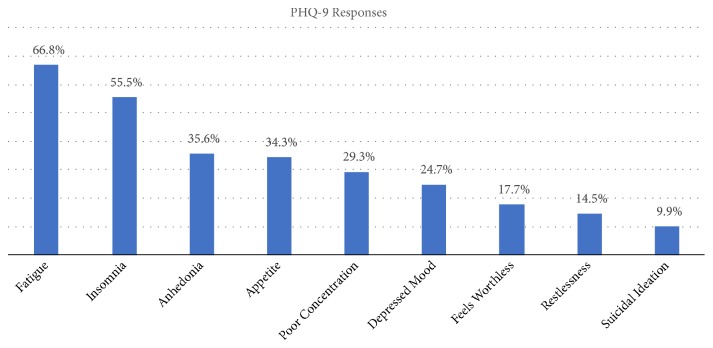
Presenting symptoms among study respondents.

PHQ 9 assessment score for depression showed that fatigue and insomnia were the most common symptoms in the participants. Suicidal ideation was present in 9.9% of participants (Figure 2).

The association of between various sociodemographic and clinical characteristics with depression is shown in Table 2. Depression was found to be significantly higher among patients who were smoking (p = 0.029) and among patients who were on insulin therapy (p = 0.026) (Table 2).

On regression analysis, both being a current smoker and being on insulin therapy were found to be independent predictors of mild to moderate depression among diabetic patients. Patients on insulin therapy were almost twice as likely to have mild to moderate depression (OR 1.78 [95% CI 1.12 – 2.82], p = 0.015). Current smokers were almost seven times more likely to have mild to moderate depression (OR 6.72 [95% CI 1.26 – 35.70], p = 0.025) (Table 3).

## 4. Discussion

Three hundred and seven participants (87%) reported to have some form of depression which was mainly of minimal and mild severity, constituting about 90% of the overall depressed patients. Only about 8% had moderate depression. None of the participants were diagnosed with severe depression. The prevalence of mild to moderate depression among diabetes patients in this study (30%) is comparable to other studies [8–10].

There is significant variation in prevalence of depression among different studies, which can be explained by the different environmental, cultural, ethnic, and social backgrounds. For example, a study done in Palestine found a high prevalence of depression, possibly due to more stressful conditions like wars, violence, and unemployment [11]. A study done in Dar es Salaam to determine common mental disorders among users of traditional healers (THC) and primary health clinics (PHC) found the prevalence of depression to be 55% among those attending THC and 48% among those attending PHC [12], which is slightly higher than the prevalence of mild to moderate depression in our study. This could be explained by the fact that they used Clinical Interview Schedule-Revised (CIS-R) as the screening tool and they had a different population than ours.

It has been suggested that diabetes (both T1DM and T2DM) is associated with increased occurrence of certain psychiatric disorders. Suicidal ideas as well as suicide attempts are potentially life-threatening psychiatric emergencies that occur more frequently in patients with DM than in the general population [13]. Many studies have focused on the relationship that DM shares with psychiatric disorders, especially depressive disorder. However, fewer studies have focused upon understanding suicidality among individuals with DM [13]. In this study 9.9% among depressed participants had suicidal thought; it is beyond the scope of this study to analyze and assess suicidality in detail.

In this study depression was significantly associated with being a current smoker and being on insulin therapy.

Patients on insulin therapy were almost twice as likely to have mild to moderate depression compared to patients who were on other therapies. This association between insulin therapy and depression has been observed in other studies done in Republic of Korea [14] and China [15]. A recently published meta-analysis also confirmed this association [16]. There is usually a negative attitude towards insulin therapy. This stems from fear of insulin self-injection, dose adjustments, weight gain, hypoglycemia, and fear of advanced disease and hence more complications. These psychological aspects can lead to depression in these patients.

Patients who were current smokers were seven times more likely to have mild to moderate depression as compared to nonsmokers. The associated between smoking and depression has been described in other studies [17, 18]. One hypothesis suggests that smoking can lead to changes in neurocircuitry which can make an individual more susceptible to environmental stressors, thus leading to depression.

## 5. Conclusions

There was high prevalence of depression in this diabetic population. Majority of patients had minimal depression but about 30% had either mild or moderate depression. Being a current smoker and being on insulin therapy were strongly associated with having mild to moderate depression. A holistic approach that focuses on the identification and management of depression among patients with diabetes is recommended.

## Figures and Tables

**Table 1:** Demographic and clinical characteristics of the study population (N = 353).

**Variable**	**Frequency**	**Percentage (**%**)**

**Age group (years)**		
Less than 20	45	12.7
21 to 40	53	15.0
41 to 60	156	44.2
61 and above	99	28.0
**Sex: Female**	226	64.9
**Marital Status**		
Single	71	20.1
Married	222	62.9
Divorced	11	3.1
Widow	49	3.9
**Education Level**		
No formal education	46	13.0
Primary School	174	49.3
Secondary School	90	25.5
College/University	43	12.2
**Occupation**		
Employed	133	37.7
Unemployed	152	43.1
Retired	68	19.3
**Current Alcohol Intake**	18	5.1
**Current Cigarette Smoking**	7	2.0
**Type of DM**		
Type 1	72	20.4
Type 2	281	79.6
**Duration since DM (years)**		
1 – 10 years	226	64.0
> 10 years	127	36.0
**On Insulin Therapy**	152	43.1
**On OHA Therapy**	254	72.0
**Body Mass Index (BMI)**		
Underweight	23	6.5
Normal	145	41.1
Overweight	105	29.7
Obese	80	22.7
**Hypertension**	166	47.0

**Table 2:** Association between depression and clinical/sociodemographic characteristics.

**Variable**	**No – Minimal Depression (**%**)**	**Mild – Moderate Depression (**%**)**	**Total**	**p-value**
	**(n = 246)**	**(n = 107)**	**(N = 353)**	

**Age group (years)**				
Less than 20	30 (66.7)	15 (33.3)	45	
21 to 40	35 (66.0)	18 (34.0)	53	
41 to 60	109 (69.9)	47 (30.1)	156	
61 and above	72 (72.7)	27 (27.3)	99	0.810
**Sex**				
Male	88 (71.0)	36 (29.0)	124	
Female	158 (69.0)	71 (31.0)	226	0.700
**Marital Status**				
Single	48 (67.6)	23 (32.4)	71	
Married	157 (70.7)	65 (29.3)	222	
Divorced	6 (54.5)	5 (45.5)	11	
Widow	35 (71.4)	14 (28.6)	49	0.677
**Education Level**				
No formal education	28 (60.9)	18 (39.1)	46	
Primary School	123 (70.7)	51 (29.3)	174	
Secondary School	64 (71.1)	26 (28.9)	90	
College/University	31 (72.1)	12 (27.9)	43	0.577
**Occupation**				
Employed	99 (74.4)	34 (25.6)	133	
Unemployed	96 (63.2)	56 (36.8)	152	
Retired	51 (75.0)	17 (25.0)	68	0.067
**Current Alcohol Intake**				
Yes	14 (77.8)	4 (22.2)	18	
No	232 (69.3)	103 (30.7)	335	0.601
**Current Cigarette Smoking**				
Yes	2 (28.6)	5 (71.4)	7	
No	244 (70.5)	102 (29.5)	346	**0.029**
**Type of DM**				
Type 1	46 (63.9)	26 (36.1)	72	
Type 2	200 (71.2)	81 (28.8)	281	0.251
**Duration since DM (years)**				
1 – 10 years	160 (70.8)	66 (29.2)	226	
> 10 years	86 (67.7)	41 (32.3)	127	0.549
**On Insulin Therapy**				
Yes	96 (63.2)	56 (36.8)	152	
No	150 (74.6)	51 (25.4)	201	**0.026**
**On OHA Therapy**				
Yes	175 (68.9)	79 (31.1)	254	
No	71 (71.7)	28 (28.3)	99	0.699
**Body Mass Index (BMI)**				
Underweight	13 (56.5)	10 (43.5)	23	
Normal	99 (68.3)	46 (31.7)	145	
Overweight	72 (68.6)	33 (31.4)	105	
Obese	62 (77.5)	18 (22.5)	80	0.222
**Hypertension**	120 (72.3)	46 (27.7)	166	0.354

**Table 3:** Multivariate analysis for predictors of mild to moderate depression among diabetic patients.

**Variable**	**OR**	**95**%** CI**	**p-value**

On Insulin Therapy	1.78	1.12 – 2.82	0.015
Current Cigarette Smoking	6.72	1.26 – 35.70	0.025

## Data Availability

The data that support the findings of this study are available from the Muhimbili University of Health and Sciences, but restrictions apply to the availability of these data, which were used under license for the current study and so are not publicly available. Data are however available from the authors upon reasonable request and with permission of Muhimbili University of Health and Allied Sciences.
